# The Effect of Ticagrelor on Endothelial Function Compared to Prasugrel, Clopidogrel, and Placebo: A Systematic Review and Meta-Analysis

**DOI:** 10.3389/fcvm.2021.820604

**Published:** 2022-01-26

**Authors:** Baoyi Guan, Lin Zhao, Dan Ma, Yixuan Fan, He Zhang, Anlu Wang, Hao Xu

**Affiliations:** ^1^Xiyuan Hospital, China Academy of Chinese Medical Sciences, Beijing, China; ^2^National Clinical Research Center for Chinese Medicine Cardiology, Beijing, China; ^3^Graduate School, Beijing University of Chinese Medicine, Beijing, China

**Keywords:** ticagrelor, endothelial function, vascular function, efficacy, meta-analysis

## Abstract

**Background/Objective:**

Endothelial dysfunction is associated with the long-term outcomes in patients with coronary artery disease (CAD). Recent evidence suggests that ticagrelor, a potent antiplatelet agent, improves endothelial function. However, several studies demonstrated contrasting results. The objective of this meta-analysis was to determine the efficacy of ticagrelor treatment on endothelial function.

**Materials and Methods:**

A systematic literature study was conducted on databases including PubMed, Web of Science, EMBASE, Scopus, and the Cochrane Library. A historical search was performed for a reference list of the selected studies as of August 2021. The randomized controlled trials (RCTs) were assessed using the Cochrane tool. The weighted mean difference (WMD) 95% CI was treated as the overall effect size, and data were pooled using the fixed-effect model or random-effect model according to the heterogeneity. Subgroup and sensitivity analyses were performed to measure the effects of potential confounders.

**Results:**

A total of 21 studies were included. The meta-analysis indicated that ticagrelor resulted in a significant increase of flow-mediated dilation (FMD) (WMD: 1.48; 95% CI: 0.36, 2.60), reactive hyperemia index (RHI) (WMD: 0.06; 95% CI: 0.00, 0.13), and circulating progenitor endothelial cells (CEPCs) (WMD: 13.84; 95% CI: 5.70, 21.98), and a reduction in the index of microvascular resistance (IMR) (WMD: −15.39; 95% CI: −25.11, −5.68).

**Conclusion:**

Ticagrelor has a significant effect on some markers of endothelial function in patients with CAD. However, the results should be interpreted with caution due to the heterogeneity and limited studies.

## Introduction

Ticagrelor is a potent direct acting, and reversibly binding P2Y12 receptor inhibitor that is recommended for the prevention of atherothrombotic events in acute coronary syndromes (ACSs) and patients with coronary artery disease (CAD) and with or without invasive management (1–3). In addition to its antiplatelet and antithrombotic actions, ticagrelor has a pleiotropic (off-target) effect primarily mediated by adenosine metabolism (4). These adenosine-mediated effects include attenuation of endothelial dysfunction associated with outcomes of CAD and are considered a potential therapeutic direction (5–7). Indeed, endothelial dysfunction is a systemic pathological change involving coronary arteries and the pathophysiological process of various disease states, including heart failure, chronic kidney disease, hypertension, and diabetes.

Several clinical trials investigated the effect of ticagrelor on endothelial function, however, these studies returned conflicting results as to whether ticagrelor improves endothelial function. Several studies suggested that ticagrelor ameliorated endothelial dysfunction (8–11). In contrast, other studies demonstrated that ticagrelor conferred no additional beneficial effect on endothelial dysfunction (5, 12, 13).

Differences in the study design, duration of intervention, study population, and methods of assessing endothelial function in these clinical trials led to inconsistent results. To date, no meta-analysis has been conducted that systematically reviewed findings from randomized controlled trials (RCTs) on the effects of ticagrelor and endothelial function. In this regard, the current meta-analysis of RCTs, based on the most comprehensive search, was performed to summarize the effects of ticagrelor on endothelial function. The common parameters of endothelial function including flow-mediated dilation (FMD), as an index of endothelium-dependent vasodilation (14); reactive hyperemia index (RHI), as an indicator of peripheral microvascular endothelial function (15); index of microvascular resistance (IMR), as an indicator of coronary microvascular endothelial function (16); and circulating progenitor endothelial cells (CEPCs), circulating endothelial cells (CECs), as the modulator of the endothelial repair processes (17, 18).

## Methods

### Search and Studies Selection Strategies

The protocol of this meta-analysis was registered at https://www.crd.york.ac.uk/PROSPERO/, ID: CRD42021259674. The following databases were searched from inception to the end of August 2021: PubMed, Web of Science, EMBASE, SCOPUS, and the Cochrane Central Register of Controlled Trials (CENTRAL). In the search strategy, the following free text search terms were used: (“ticagrelor” [Mesh] OR (Brilique [Title/Abstract] OR AZD6140 [Title/Abstract] OR Brilinta [Title/Abstract]) AND (endothelial [Title/Abstract] OR vascular [Title/Abstract]). The lists of references were scrutinized to identify articles of interest that were not included in the original search.

### Eligibility Criteria

We included all the trials with randomized, controlled, parallel, or cross-over designs that analyzed the effects of ticagrelor administration on endothelial function. Control groups receiving clopidogrel or prasugrel were used. Other studies, such as review articles, animal experiments, cell culture studies, *in vitro* studies, trials without a control group, and studies from which we could not extract data, were excluded.

### Data Collection

Authors (BY Guan and L Zhao) independently evaluated the included articles and extracted data, and any discrepancies were resolved by discussion and consensus. From each eligible study, the following data were extracted based on a standardized extraction form: name of first author, year of publication, country, study design, sample size, age, dosage, study duration, the mean and SD for FMD, RHI, IMR, CEPCs, and circulating endothelial cells (CECs) in each group.

### Risk of Bias Assessment

The risk of bias in the included RCTs was assessed using the Cochrane Collaboration risk of the bias tool based on the following criteria: “randomization process, allocation concealment, blinding of participants and outcome evaluator, incomplete outcome data, and selective outcome reporting, and other potential sources of bias.” The Egger regression test and the Begg-Mazumdar correlation test were used to reveal evidence of publication bias.

### Data Synthesis and Statistical Analysis

Changes in FMD, RHI, IMR, CEPCs, and CECs, were used to assess the effect of ticagrelor administration on these outcomes determining the difference between the intervention and control groups with mean and SD. Continuous variables were used to analyze the weighted mean difference (WMD) with the 95% CI effect size. Cochrane's *Q* test combined with the *P*-value (at the <0.10 level was considered significant) and chi-squared test were used to evaluate the heterogeneity among studies. The chi-squared statistic varying from 0 to 100% was used to specify the expanse of heterogeneity, and *I*^2^ > 50% was considered high heterogeneity. We used the fixed-effect model or the random-effects model in the meta-analysis according to the chi-squared values. Subgroup analyses investigated the type of control drug, duration of treatment, study design, population, sample size, and age. STATA 12.0 (Stata Corp., College Station, TX, USA) and Review Manager 5.3 (Cochrane Collaboration, Oxford, UK) were used for data analyses.

## Results

### Study Characteristics

**Figure 1:**
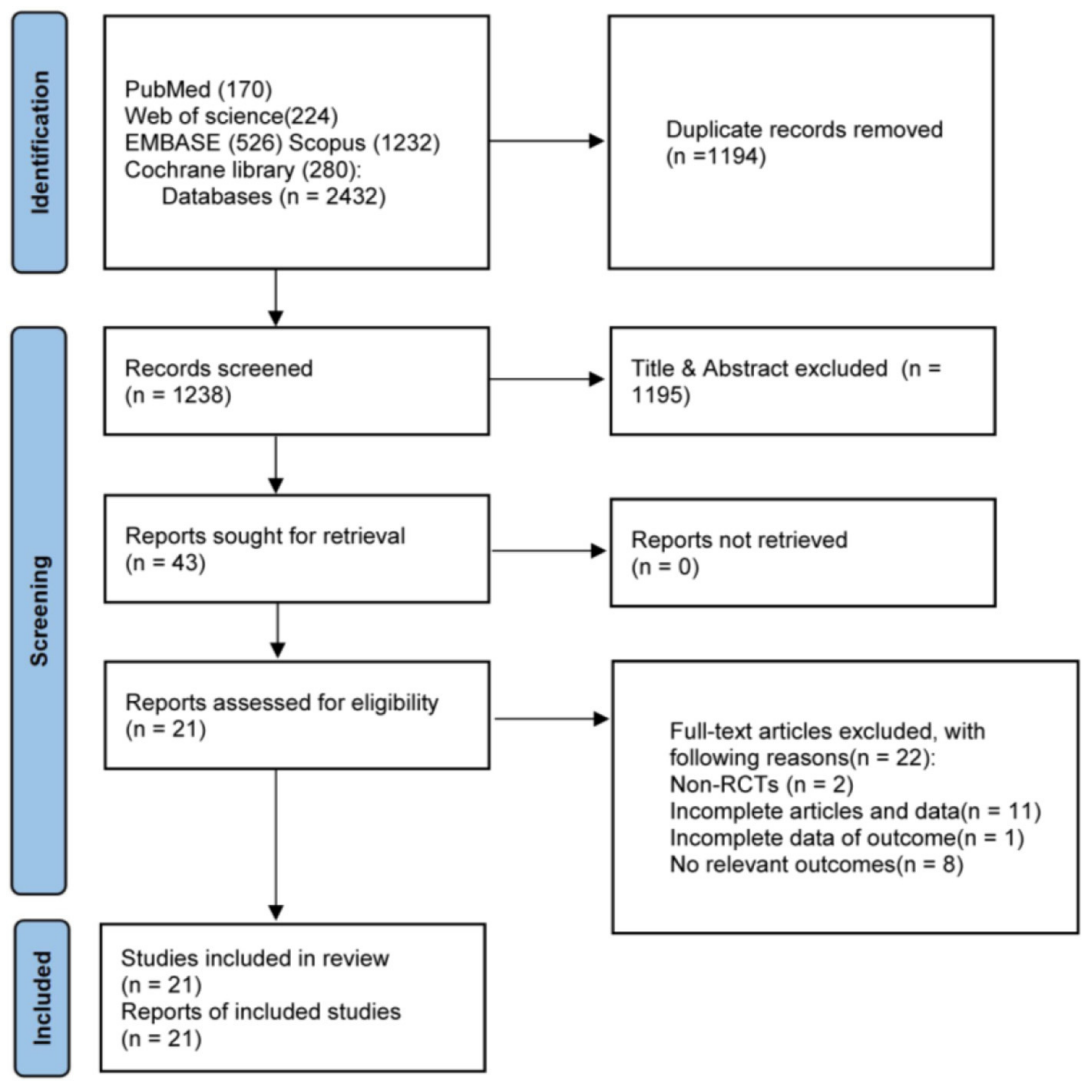
Literature search and review flowchart for selection of studies.

A total of 21 studies involving 1,746 participants were eligible for this meta-analysis. The flow diagram of the studies selected is presented in Figure 1. These studies were published between 2014 and 2021. All the studies were RCTs. Detailed characteristics of included studies are provided in Table 1. Of these, four studies had a cross-over design, and the others had a parallel design. The participants of the studies consisted of subjects mainly with CAD (i.e., stable CAD, unstable angina (UA), non-ST elevation myocardial infarctions (ACS), with or without stent implantation). According to the intervention, four studies assessed the effects of ticagrelor compared with prasugrel and clopidogrel, and others only compared with either agent. One study evaluated various doses of ticagrelor. Of the 21 included primary studies, nine reported the difference of FMD after ticagrelor administration, five studies reported the difference of RHI, five studies reported the difference of IMR, and three studies reported the difference of CEPCs or CECs.

**Table 1:** Characteristics of included studies.

**References**	**Country/population**	**Sample size and intervention**	**Age (y) (corresponding to intervention)**	**Duration**	**Presented data**
Schnorbus et al. (19)	Germany/patients with UA or NSTEMI undergoing coronary intervention	16, ticagrelor (180 mg loading dose, 90 mg twice daily), 31, clopidogel (600 mg loading dose, 75 mg once daily)	60.5 ± 9.0 62.2 ± 10.3	1 month	FMD
Schnorbus et al. (19)	Germany/patients with UA or NSTEMI undergoing coronary intervention	17, ticagrelor (180 mg loading dose, 90 mg twice daily), 27, prasugrel (60 mg loading dose, 10 mg once daily)	60.5 ± 9.0 60.6 ± 7.8	1 month	FMD
He et al. (20)	China/patients with stable CAD	15, ticagrelor (22.5 mg b.i.d.), 15, ticagrelor (45 mg b.i.d.), 15, ticagrelor (90 mg b.i.d.), 15, clopidogrel (75 mg o.d.)	63.4 ± 6.93, 64.07 ± 6.03, 64.53 ± 7.16, 65.4 ± 6.01	28 days	FMD
Lim et al. (12)	Korea/patients with NSTEACS	20, ticagrelor (90 mg b.i.d.), 20, clopidogrel (75 mg o.d.)	65.3 ± 9.6, 61.9 ± 11.2	30 days	FMD
Ariotti et al. (5)	Europe/stable post-ACS patients	4, ticagrelor (180 mg loading dose, 90 mg twice daily), 9, clopidogel (600 mg loading dose, 75 mg once daily)	60.1 ± 10.6, 64.9 ± 8.1	30 ± 5 days	FMD, RHI
Ariotti et al. (5)	Europe/stable post-ACS patients	5, ticagrelor (180 mg loading dose, 90 mg twice daily), 9, prasugrel (60 mg loading dose, 10 mg once daily)	60.1 ± 10.6, 60.8 ± 12.20,	30 ± 5 days	FMD, RHI
Jeong et al. (10)	Korea/type 2 diabetic patients with NSTEACS requiring stent implantation	60, ticagrelor (180 mg loading dose, 90 mg twice daily), 61, prasugrel (60 mg loading dose, 10 mg once daily)	62.0 ± 9.2 60.2 ± 9.2	10 weeks	FMD, CEPCs
Xu et al. (21)	Australia/NSTEACS patients	36, ticagrelor (180 mg loading dose, 90 mg twice daily), 33, clopidogel (600 mg loading dose, 75 mg once daily)	59 (IQR 51–58.8)	–	FMD
Xu et al. (22)	Australia/NSTEACS patients	45, ticagrelor (180 mg loading dose, 90 mg twice daily), 43, clopidogel (600 mg loading dose, 75 mg once daily)	59 (IQR 51–58.8)	–	IMR
Mangiacapra et al. (23)	Italy/type 2 diabetes mellitus and stable CAD treated with PCI and drug-eluting stent implantation	21, ticagrelor (90 mg twice daily), 21, clopidogel (150 mg once daily)	–	14 days	FMD
Siasos et al. (24)	Greece/stable CAD	7, ticagrelor (90 mg twice daily), 15, clopidogel (75 mg once daily)	54 ± 11 55 ± 8	1 month	FMD
Siasos et al. (24)	Greece/stable CAD	8, ticagrelor (90 mg twice daily), 15, prasugrel (10 mg once daily)	54 ± 11, 58 ± 10	1 month	FMD
Park et al. (25)	Korea/patients with ACS requiring stent implantation	54, ticagrelor (180 mg loading dose, 90 mg twice daily), 53, clopidogel (600 mg loading dose, 75 mg once daily)	56.9 ± 11.4, 58.5 ± 9.9	12 months	IMR
Choi et al. (9)	Korea/patients with non-significant coronary disease	41, ticagrelor (90 mg twice daily or 90 mg twice daily) 20, clopidogrel (75 mg once daily)	60.95 ± 8.68, 66.85 ± 8.52	7 days	IMR
van der Hoeven et al. (8)	Netherlands and Spain/patients with STEMI	53, ticagrelor (180 mg loading dose, 90 mg twice daily), 51, prasugrel (60 mg loading dose, 10 mg once daily)	60.1 ± 10.4, 61.2 ± 8.8	1 year	RHI
Xanthopoulou et al. (26)	Greece/patients with stable CAD	11, ticagrelor (90 mg twice daily), 11, prasugrel (10 mg once daily)	55.5 ± 8.8, 59.8 ± 6.7	15 days	RHI
Diego-Nieto et al. (13)	Spain/NSTEMI patients	47, ticagrelor (180 mg loading dose, 90 mg twice daily), 49, clopidogel (600 mg loading dose, 75 mg once daily)	65.6, 67.7	1 month	CEPCs, CECs
Tatsidou et al. (27)	Greece/ACS patients	31, ticagrelor (180 mg loading dose, 90 mg twice daily), 36, clopidogel (600 mg loading dose, 75 mg once daily)	63 ± 11, 61 ± 13	5 days	CEPCs
Chen et al. (28)	China/ACS patients	93, ticagrelor (90 mg twice daily), 93, clopidogel (75 mg once daily)	62.57 ± 10.03, 64.82 ± 11.75,	6 months	RHI
Lobo et al. (29)	Ireland/CAD Patients	31, ticagrelor (90 mg twice daily), 31, clopidogel (75 mg once daily)	–	1 month	RHI
Oikonomou et al. (30)	Greece/patients with stable CAD	9, ticagrelor (90 mg twice daily), 34, clopidogel (75 mg once daily)	53 ± 11, 58 ± 8	1 month	FMD
Oikonomou et al. (30)	Greece/patients with stable CAD	10, ticagrelor (90 mg twice daily), 15, prasugrel (10 mg once daily)	53 ± 11, 58 ± 10	1 month	FMD
Liang et al. (31)	China/UAP patients	73, ticagrelor (90 mg twice daily), 73, clopidogel (75 mg once daily)	66.7 ± 5.7, 68.1 ± 6.6	12 months	circulating ECs
Wang et al. (32)	China/CHD patients with confirmed type 2 diabetes	72, ticagrelor (90 mg twice daily), 72, clopidogel (75 mg once daily)	–	30 days	CECs
Park et al. (33)	South Korea/Patients with STEMI	38, ticagrelor (180 mg loading dose), 38, clopidogel (600 mg loading dose)	–	Receive a loading dose before primary PCI	IMR
Choi et al. (9)	South Korea/patients with CAD	12, ticagrelor (180 mg loading dose), 12, clopidogel (600 mg loading dose)	–	–	IMR

*UA, unstable angina; NSTEMI, non-ST elevation myocardial infarctions; CAD, coronary artery disease; ACS, acute coronary syndromes; NSTEACS, non-ST segment elevation acute coronary syndromes; STEMI, ST-elevation myocardial infarctions; UAP, unstable angina pectoris; CHD, coronary heart disease; PCI, percutaneous coronary intervention; FMD, flow-mediated dilation; RHI, reactive hyperemia index; IMR, index of microvascular resistance; CEPCs, circulating progenitor endothelial cells; CECs, circulating endothelial cells*.

### Risk of Bias Assessment

**Figure 2:**
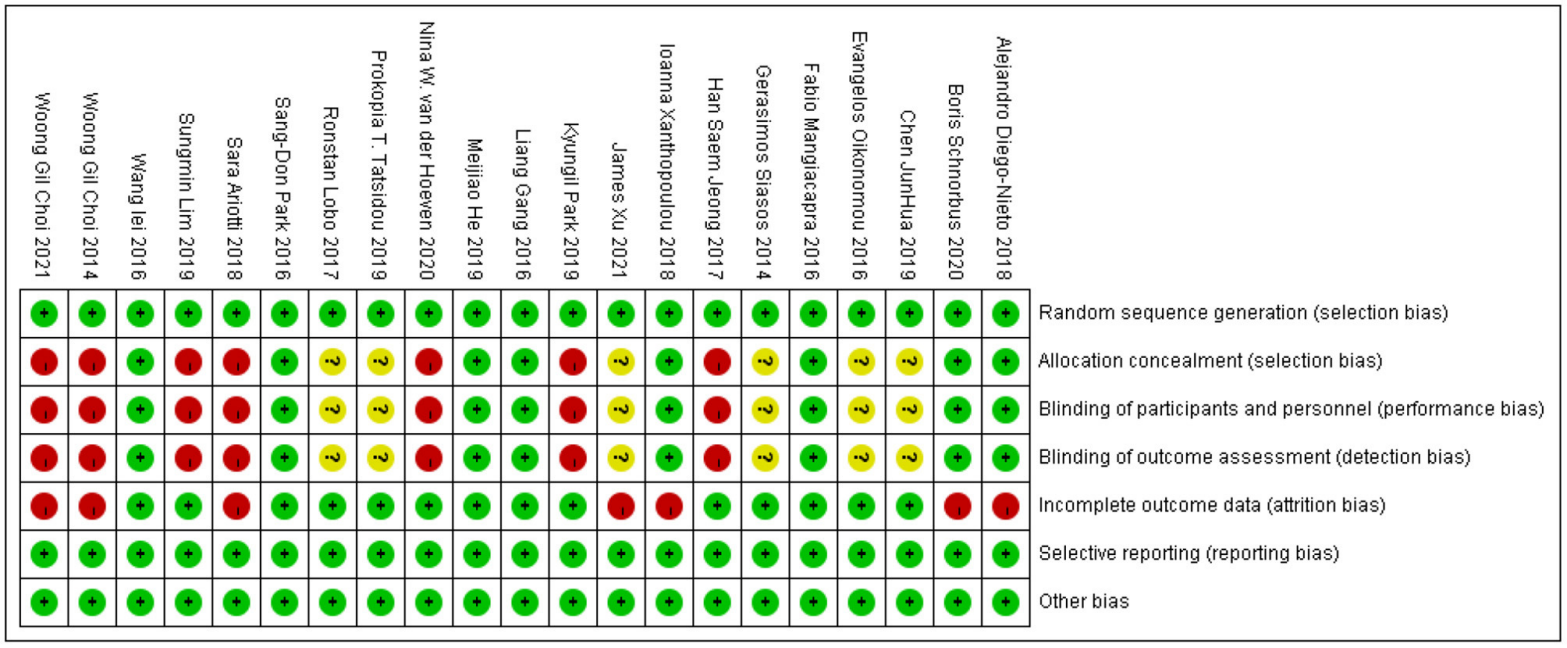
Risk of bias assessment of the included studies.

The risk of bias assessments is shown in Figure 2. In six of the 21 studies, there was an unclear risk of bias for selection in the domains of allocation concealment and an unclear risk of bias for implementation and measurement in terms of blinding of participants and personnel, blinding of outcome assessment. In the risk of bias assessment aforementioned, seven studies had a high risk of bias judgment. In addition, seven studies lacked information or outcome data and possessed a high risk of bias.

### Meta-Analysis

#### Effect of Ticagrelor on FMD

**Figure 3:**
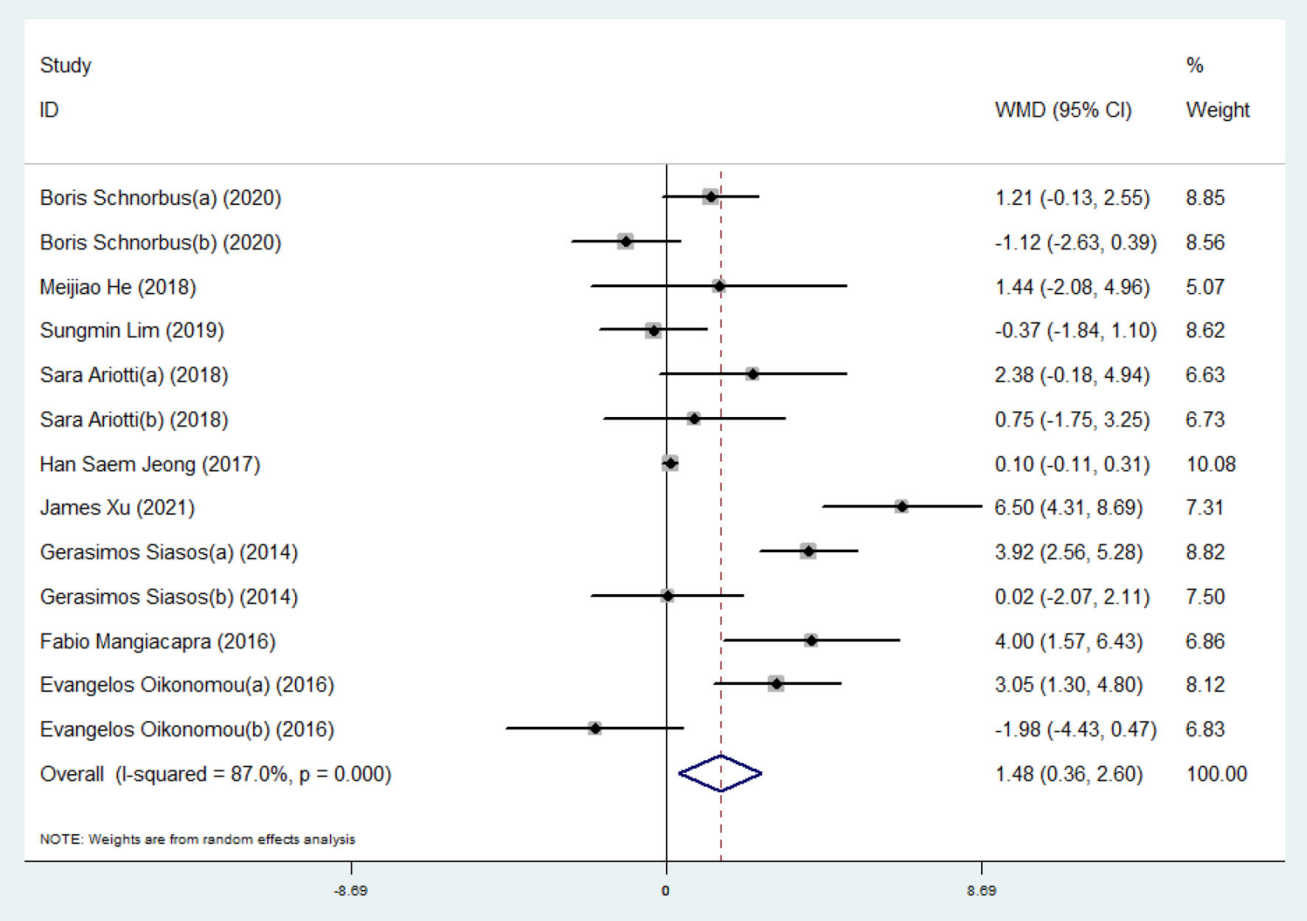
A pooled estimate of ticagrelor effect on flow-mediated dilation.

A meta-analysis of 13 effect sizes from nine studies (5, 10, 12, 19–21, 23, 24, 30) (258 participants in the ticagrelor group and 305 participants in the control group) showed that ticagrelor administration gave rise to significantly higher FMD (WMD: 1.48; 95% CI: 0.36, 2.60) (Table 2 and Figure 3) than a control group using the results of the randomized-effect model. Several subgroup analyses were performed to explore heterogeneity and determine the influence of factors on the estimated effect size. Analysis of the administration of the control group showed that ticagrelor administration caused a notable increase in FMD compared with the clopidogrel control (WMD: 2.74; 95% CI: 1.21, 4.28), but not to the prasugrel control (WMD: −0.21; 95% CI: −0.90, 0.49) (Table 3 and Supplementary Figure 1). Analysis of the study population subgroups revealed that ticagrelor was associated with a greater increase in FMD in Caucasians (WMD: 1.88; 95% CI: 0.33, 3.42) than East Asians (WMD: 0.10; 95% CI: −0.12, 0.31) (Table 3 and Supplementary Figure 2). When the included studies were stratified in two subgroups based on the study design (parallel and cross-over), subgroup analyses showed similar changes in the increase of FMD following ticagrelor administration (parallel, WMD: 1.41; 95% CI: −0.25, 3.08; cross-over, WMD: 1.60; 95% CI: −0.31, 3.51), but not significantly (Table 3 and Supplementary Figure 3). In the view of study sample size ( ≤ 50 and > 50), changes in FMD did not reach significant levels when the sample size ≤ 50 (WMD: 1.19; 95% CI: −0.13, 2.50) compared with the sample size > 50 (WMD: 2.65; 95% CI: −1.80, 7.11) (Table 3 and Supplementary Figure 4). As for age of the participants (≤ 60 and > 60 years), the increasing effect of ticagrelor on FMD in participants with age ≤ 60 years (WMD: 2.37; 95% CI: −0.16, 4.89) was greater than in subjects with age > 60 years (WMD: 0.26; 95% CI: −0.40, 0.91) (Table 3 and Supplementary Figure 5).

**Table 2:** The effects of ticagrelor on markers of endothelial function of included studies.

**Outcomes**	**Number of effect sizes**	**Treatment effect**		**Heterogeneity**	
		**WMD**	**95% CI**	**I2 (%)**	***P*-value**
FMD	13	1.48	0.36, 2.60	87.0	<0.001
RHI	6	0.06	0.00, 0.13	0	0.679
IMR	5	−15.39	−25.11, −5.68	87.0	<0.001
CEPCs	3	13.84	5.70, 21.98	98.5	<0.001
CECs	3	−1.08	−8.63, 6.47	85.8	0.001

**Table 3:** Subgroup analyses for the effects of ticagrelor on markers of endothelial function of included studies.

**Outcomes**		**Subgroups**	**Number of effect sizes**	**Treatment effect**		**Heterogeneity**		***P*-value (between group)**
				**Pooled WMD**	**95% CI**	***I*^2^ (%)**	***P*-value**	
FMD	Controlled administration	Clopidogrel prasugrel	8 5	2.74 −0.21	1.21, 4.28 −0.90, 0.49	81.3 26.6	<0.001 0.244	<0.001
	Study design	Parallel Cross-over	9 4	1.41 1.60	−0.25, 3.08 −0.31, 3.51	87.3 76.9	<0.001 0.005	0.89
	Study population	Caucasian Eastern Asian population	10 3	1.88 0.10	0.33, 3.42 −0.12, 0.31	85.1 0	<0.001 0.624	0.03
	Study sample size	≤ 50 > 50	10 3	1.19 2.65	−0.13, 2.50 −1.80, 7.11	80.8 94.0	<0.001 <0.001	0.54
	Participants' age	≤ 60 > 60 Not reported	5 7 1	2.37 0.26 ...	−0.16, 4.89 −0.40, 0.91 ...	88.6 36.2 ...	<0.001 0.152	0.005
RHI	Control administration	Clopidogrel prasugrel	3 3	0.06 0.04	−0.05, 0.18 −0.05, 0.14	0 0	0.306 0.82	0.77
	Study design	Parallel Cross-over	3 3	0.08 −0.04	0.01, 0.15 −0.22, 0.13	0 0	0.598 0.76	0.21
	Study duration	> 1 month ≤ 1 month	2 4	0.08 −0.03	0.01, 0.15−0.19, 0.12	0 0	0.4 0.901	0.17

#### Effect of Ticagrelor on RHI

**Figure 4:**
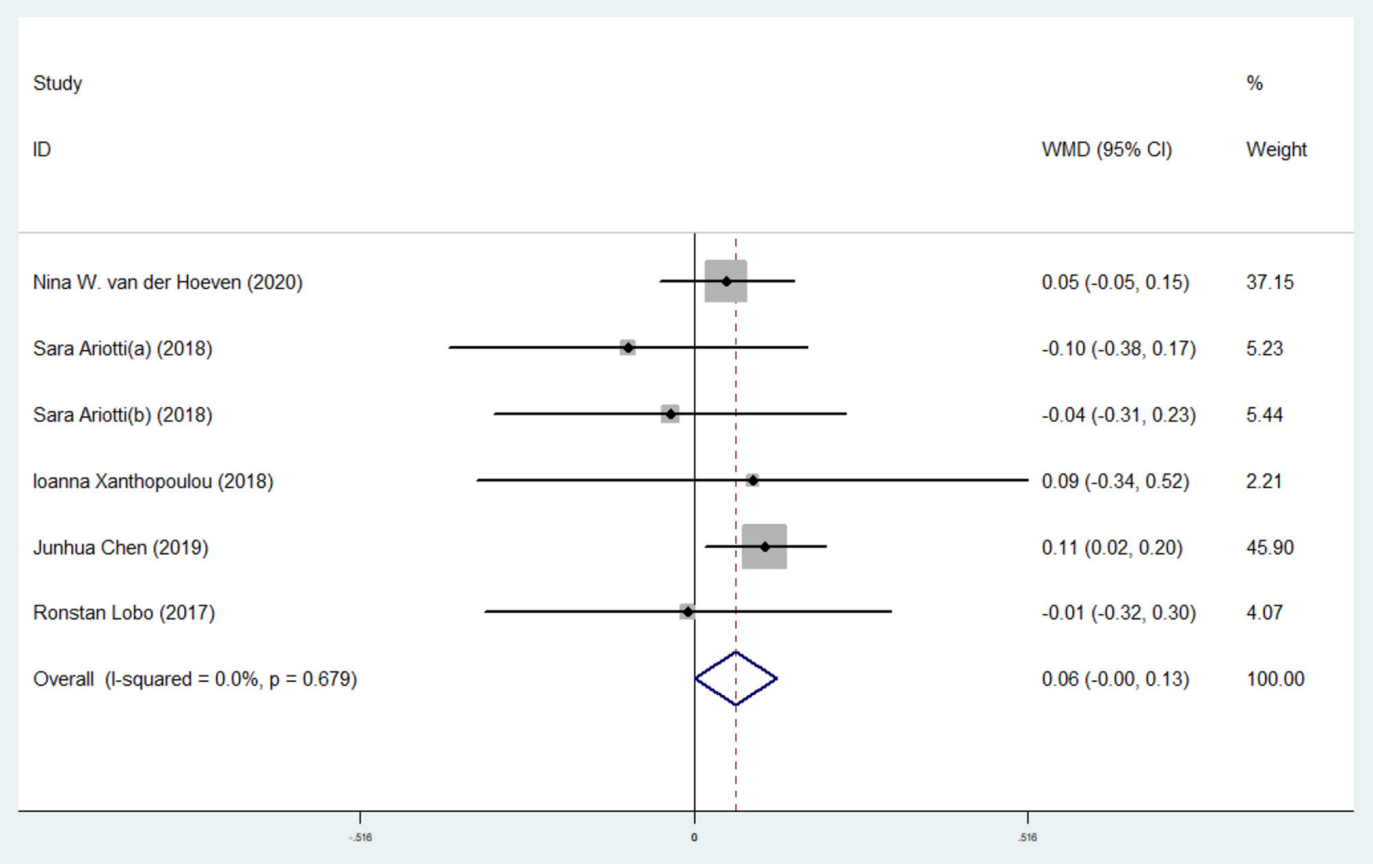
A pooled estimate of ticagrelor effect on reactive hyperemia index.

The efficacy of ticagrelor administration on RHI was investigated in five studies with six effect sizes (5, 8, 26, 28, 29). The pooled estimates revealed that ticagrelor administration substantially increased RHI compared with the control group (WMD: 0.06; 95% CI: 0.00, 0.13) (Table 2 and Figure 4). Due to the considerable heterogeneity between studies, the effects of suspected variables, including the administration of control group, study design, sample size, and study duration, were the source of heterogeneity, and subgroup analyses were performed. Regarding the results of the administration of the control group, elevating effect of ticagrelor on RHI was greater when compared with the prasugrel control (WMD: 0.04; 95% CI: −0.05, 0.14) but failed to reach a significant level as compared with the clopidogrel control (WMD: 0.06; 95% CI: −0.05, 0.18) (Table 3 and Supplementary Figure 6). As expected in the study design subgroup, the heterogeneity was reversed in subjects assigned to cross-over studies. In this subgroup analysis, changes in RHI following ticagrelor administration were not significant in cross-over studies (WMD: −0.04; 95% CI: −0.22, 0.13) compared with the parallel ones (WMD: 0.08; 95% CI: 0.01, 0.15) (Table 3 and Supplementary Figure 7). Furthermore, the result of study duration subgroup analysis showed a significant increase in RHI in individuals who received > 1 month of ticagrelor (WMD: 0.08; 95% CI: 0.01, 0.15) compared with those with ≤ 1 month of administration (WMD: −0.03; 95% CI: −0.19, 0.12) (Table 3 and Supplementary Figure 8).

#### Effect of Ticagrelor on IMR

**Figure 5:**
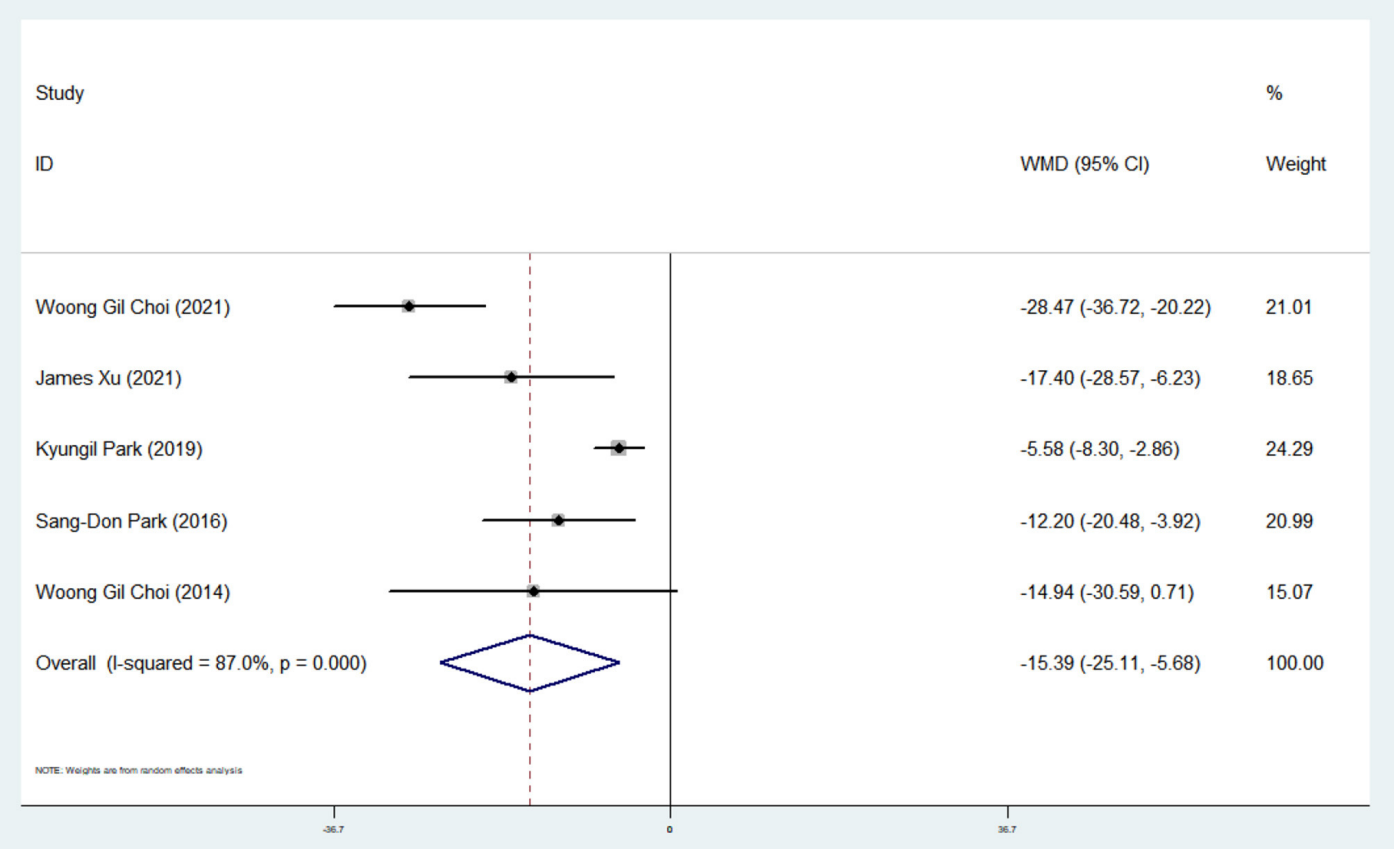
A pooled estimate of ticagrelor effect on the index of microvascular resistance.

Pooling data from five studies (9, 21, 25, 33, 34) showed a significant reduction in IMR after ticagrelor administration (WMD: −15.39; 95% CI: −25.11, −5.68) (Table 2 and Figure 5).

#### Effect of Ticagrelor on CECs

**Figure 6:**
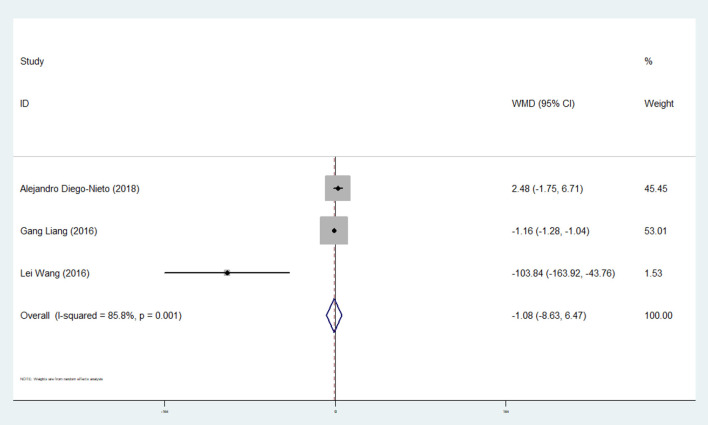
A pooled estimate of ticagrelor effect on circulating endothelial cells.

Three studies analyzed CECs with 386 participants (13, 31, 32). A meta-analysis based on changes in ticagrelor and control groups indicated that ticagrelor did produce any significant effects on CEC levels (WMD: −1.08; 95% CI: −8.63, −6.47) (Table 2 and Figure 6).

#### Effect of Ticagrelor on CEPCs

**Figure 7:**
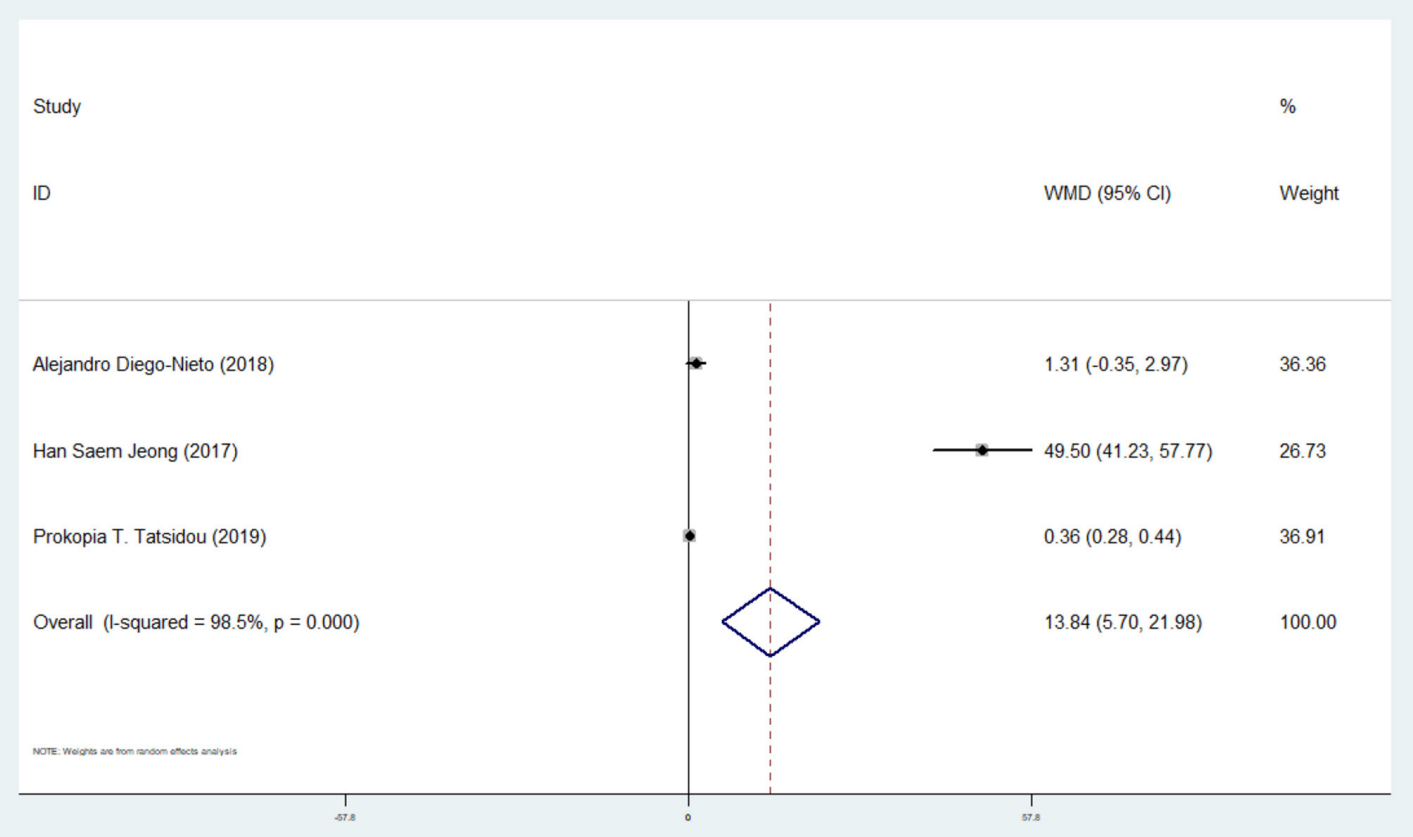
A pooled estimate of ticagrelor effect on circulating progenitor endothelial cells.

The combined analysis of three studies (10, 13, 27) showed a substantial increase in CEPCs after ticagrelor administration (WMD: 13.84; 95% CI: 5.70, 21.98) (Table 2 and Figure 7).

### Publication Bias

Egger's and Begg's tests were used to determine whether there was publication bias. Statistical Egger's test indicated no significant publication bias for the effect of ticagrelor on FMD (*P* = 0.076), RHI (*P* = 0.145), and IMR (*P* = 0.14) in the overall analysis (Supplementary Figures 9–11). The Begg-Mazumdar correlation test confirmed that there was no evidence of publication bias for FMD (Kendall's Score = 10, continuity-corrected *z* = 0.55, continuity-corrected *P* = 0.583), RHI (Kendall's Score = −3, continuity-corrected *z* = 0.38, continuity-corrected *P* = 0.707) and IMR (Kendall's Score = 0, continuity-corrected *z* = −0.24, continuity-corrected *P* = 1.000).

### Sensitivity Analyses

Based on the sensitivity analysis results, eliminating each study one at a time sequentially did not substantially influence any of the assessed factors.

## Discussion

This meta-analysis was the first to comprehensively study effect of ticagrelor on endothelial functions. Thirty effect sizes were analyzed among the 21 eligible studies. The meta-analysis of RCTs found that ticagrelor significantly increased FMD, RHI, and CEPCs, and significantly reduced IMR but did not affect CEC levels. However, only three studies reported ticagrelor-related alterations in CEC levels, which substantially reduced the statistical power. To the best of our knowledge, most included studies were carried out in Europe and eastern Asia, which increases the possibility of selection bias. Therefore, the results of this meta-analysis may not be generalizable to patients in other geographic regions.

Furthermore, heterogeneity regarding controlled administration, study design, study sample size, age of the participants, and study duration was high among the studies included in both the meta-analyses. The significance of the effect of ticagrelor disappeared in some subgroup analyses. We observed that the increasing effect was greater in FMD and RHI for ticagrelor than clopidogrel; however, these alterations were not consistent compared with the prasugrel. In this regard, some studies demonstrated that prasugrel but not clopidogrel or ticagrelor improved FMD (19, 35). Data based on the study population subgroup analysis revealed an opposite finding regarding FMD and IMR. Previous studies demonstrated that race independently influences the efficiency of ticagrelor (36, 37). Our results should be examined with caution.

Interestingly, we observed that the effect of ticagrelor on FMD and IMR was more significant in those aged ≤ 60 years than those aged > 60 years. This may be because aging impairs vasodilatory function and increases the risk of endothelial dysfunction (38, 39). Therefore, the reliability of the obtained results should be interpreted with caution because of the small number of studies and patients in each subgroup. In this regard, we used a random-effects model and conducted a sensitivity analysis to minimize the influence of heterogeneity. In addition, differences in baseline levels of markers related to endothelial function and the timing of assessment should also be considered in all these clinical studies, which may account for these conflicting results. In addition, ticagrelor and statins have been shown to exert synergistic protective effects in the pathogenesis and outcomes of CAD. Weisshaar et al. reported a clinical trial of atorvastatin combined with ticagrelor to prevent endothelial dysfunction after acute vascular occlusion compared with ticagrelor alone (40). Data on whether to use statins were also not fully attainable in the included trials; hence, we cannot offer a definitive conclusion.

^⋆⋆⋆⋆^Almost all the conventional risk factors of atherosclerosis, including obesity, hypertension, insulin resistance, and diabetes, are related to endothelial dysfunction, implying that the presence and the extent of endothelial dysfunction are associated with the prediction of subsequent cardiovascular event risk and outcome (41). Many cardiovascular pharmacotherapies, including traditional lipid-lowering agents, antihypertensive agents, and antiplatelet agents, are used partly because of their benefits against endothelial dysfunction (22). Ticagrelor, a potent antiplatelet agent, was reported to improve endothelial function. Lavi et al. found that short-term administration of ticagrelor significantly improved microvascular endothelial function in patients with CAD (42). After 1 month of taking ticagrelor, there were significantly increased levels of circulating EPCs, suggesting a benefit on vascular healing and endothelial homeostasis in ACS patients (43). However, as already mentioned, intake of standard doses of ticagrelor in healthy subjects did not improve ischemia-reperfusion induced endothelial dysfunction (44), suggesting that the potential beneficial effects of ticagrelor may only exist in patients with identified endothelial dysfunction.

Furthermore, there is a lack of evidence for deterioration in endothelial function following ticagrelor treatment cessation (45). These differences in studies may partly be due to the effects of ticagrelor administration on indicators of endothelial function may be affected by factors such as duration of intervention and participant characteristics. One hypothesis states that ticagrelor exerts vasoprotective effects by indirectly blocking adenosine phosphate receptors in addition to inhibition of platelet aggregation (46). Adenosine, a naturally occurring endogenous purine nucleotide, plays a crucial role in the endothelial cytoprotection of ticagrelor against hypoxia (6). Interestingly, it was suggested that ticagrelor might influence microvascular function through the platelet-endothelial pathway and the anti-inflammatory pathway (5, 47). This is because adenosine released by endothelial cells during ischemia and hypoxia can inhibit platelet aggregation by inhibiting internal calcium mobilization and external calcium influx (48), in turn inhibiting the release of endothelium-related inflammatory factors (49).

Moreover, nitric oxide (NO) produced by endothelial cells was first recognized as a significant vasodilator involved in controlling vasomotor function and local blood flow. Thus, endothelial dysfunction is defined as the imbalance of NO bioavailability that depends on the synthesis and metabolism of NO and the sensitivity of target tissues to NO (50). Ticagrelor inhibits the equilibrative nucleoside transporter-1 and adenosine cell re-uptake (4), thereby contributing to the endothelial release of NO (51). Understanding the mechanism of ticagrelor on endothelial function is of particular interest in terms of efficacy and adverse events.

There are several limitations to this meta-analysis. First, most of the eligible RCTs included a relatively small study population; hence the impact of confounders (i.e., sex and treatment strategies) on the findings could not be evaluated. Second, the doses of ticagrelor used differed, which may be a possible confounder that affects absorption and bioavailability. Third, although Egger's and Begg's tests showed no publication bias, the heterogeneity of the studies cannot be ignored because of the variables such as duration, control group setting, and study design. Nevertheless, the sensitivity analysis ensured the reliability of this meta-analysis. Historically, the benefits of ticagrelor were understood to be driven by the significantly lower rates of myocardial infarction and vascular death. Due to the limitations of studies, we did not study the prognostic value of endothelial dysfunction in patients with CAD. Future studies should attempt to determine the effect of ticagrelor on endothelial function.

## Conclusion

Ticagrelor can improve endothelial function by significantly increasing FMD, RHI, and CEPCs, and significantly reducing IMR. These results should be interpreted with caution because of the limited number of studies.

## Data Availability Statement

The original contributions presented in the study are included in the article/Supplementary Material, further inquiries can be directed to the corresponding authors.

## Author Contributions

All authors listed have made a substantial, direct, and intellectual contribution to the work and approved it for publication.

## Funding

This study was supported by the project of the Innovation Team and Talents Cultivation Program of National Administration of Traditional Chinese Medicine (Grant No. ZYYCXTD-C-202007) and China Academy of Chinese Medical Sciences Innovation Fund (CACMS Innovation Fund) (Grant No. CI2021A00917).

## Conflict of Interest

The authors declare that the research was conducted in the absence of any commercial or financial relationships that could be construed as a potential conflict of interest.

## Publisher's Note

All claims expressed in this article are solely those of the authors and do not necessarily represent those of their affiliated organizations, or those of the publisher, the editors and the reviewers. Any product that may be evaluated in this article, or claim that may be made by its manufacturer, is not guaranteed or endorsed by the publisher.
