# Supplementary material for: The Effect of Ticagrelor on Endothelial Function Compared to Prasugrel, Clopidogrel, and Placebo: A Systematic Review and Meta-Analysis
Source: Front Cardiovasc Med. 2022 Jan 26;8:820604. doi: 10.3389/fcvm.2021.820604 (PMC8826068; doi:10.3389/fcvm.2021.820604)
Supplement: Supplementary file 1 [file Data_Sheet_1.doc]

**Supplementary material**

**Supplemental Figure 1: Pooled estimate of ticagrelor effect on flow-mediated dilation by subgroup according to controlled administration**


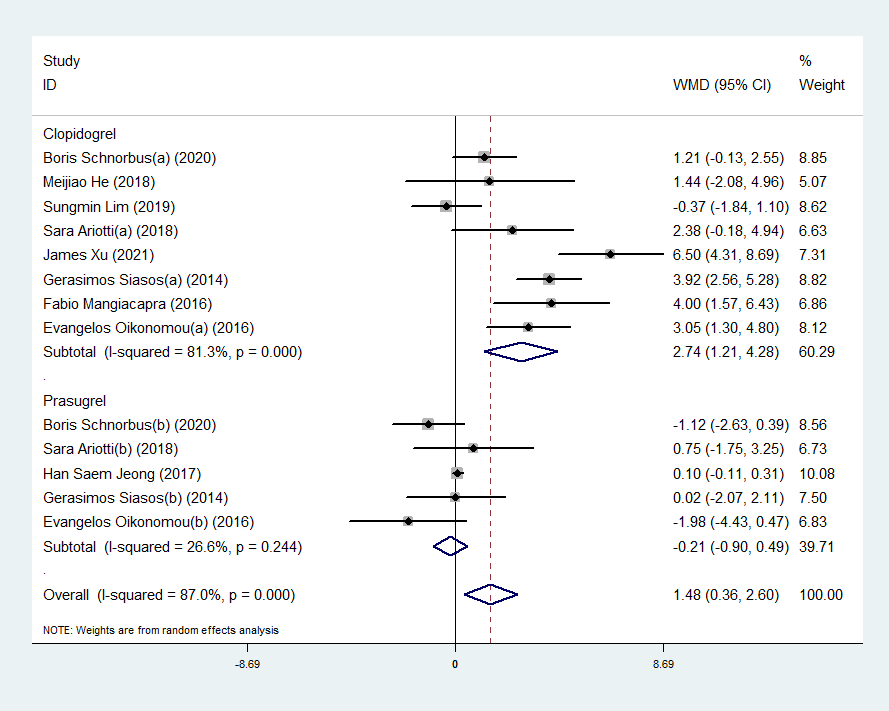


**Supplemental Figure 2: Pooled estimate of ticagrelor effect on flow-mediated dilation by subgroup according to study population**


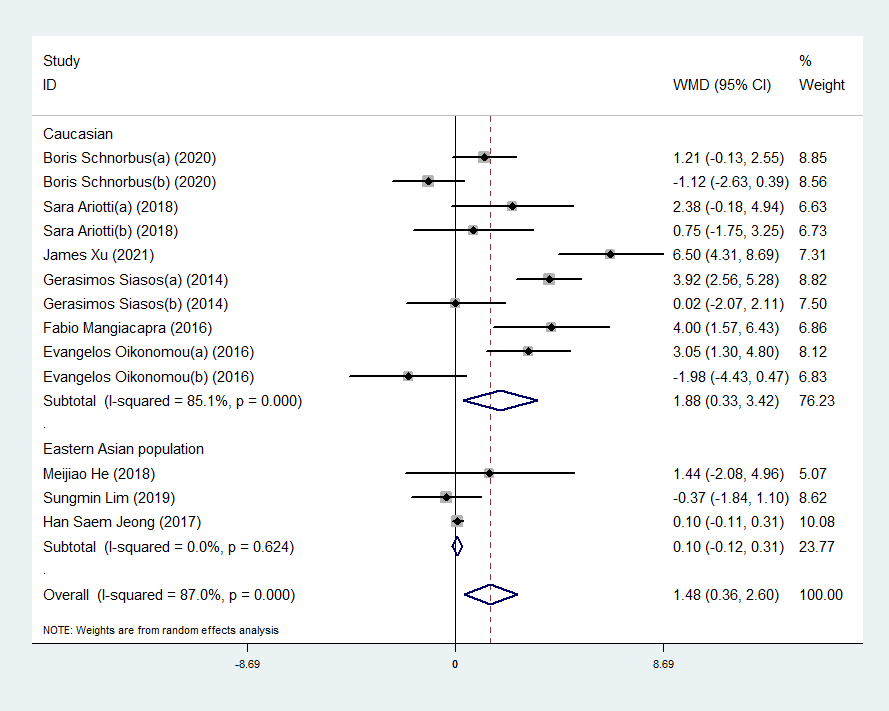


**Supplemental Figure 3: Pooled estimate of ticagrelor effect on flow-mediated dilation by subgroup according to study design**


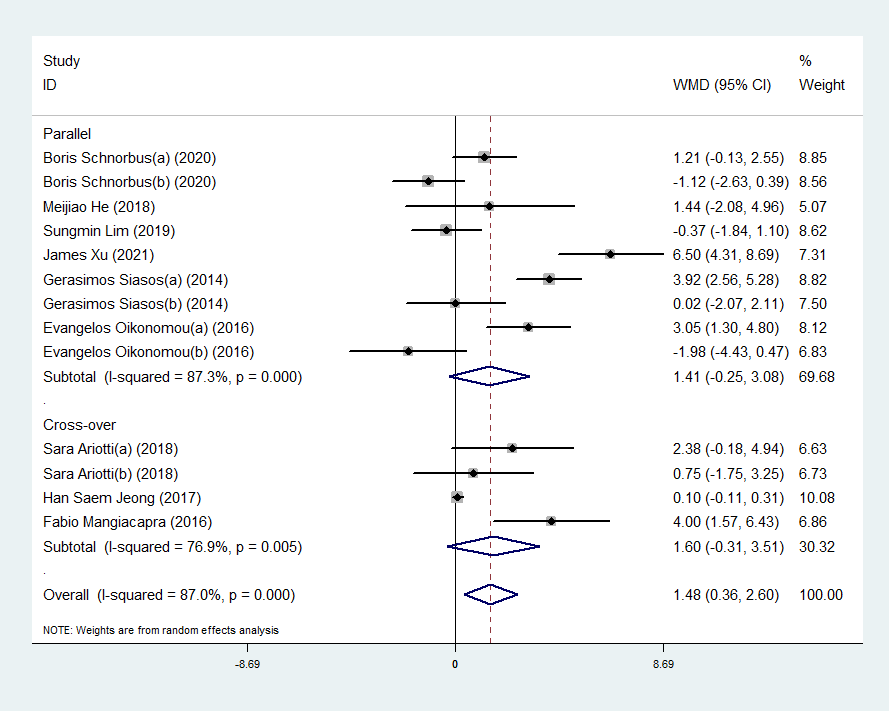


**Supplemental Figure 4: Pooled estimate of ticagrelor effect on flow-mediated dilation by subgroup according to study sample size**


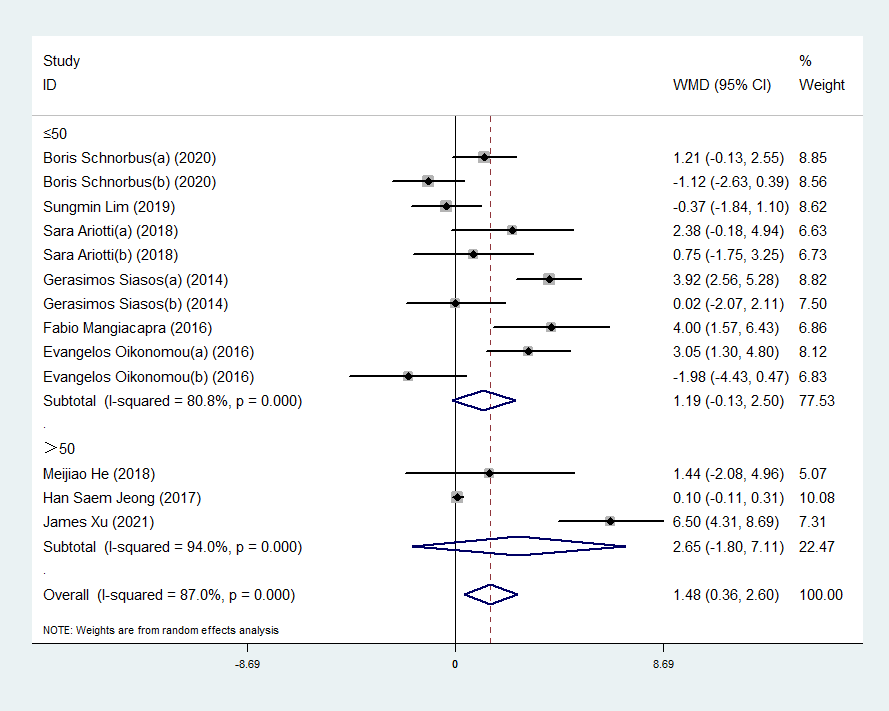


**Supplemental Figure 5: Pooled estimate of ticagrelor effect on flow-mediated dilation by subgroup according to participants’ age**


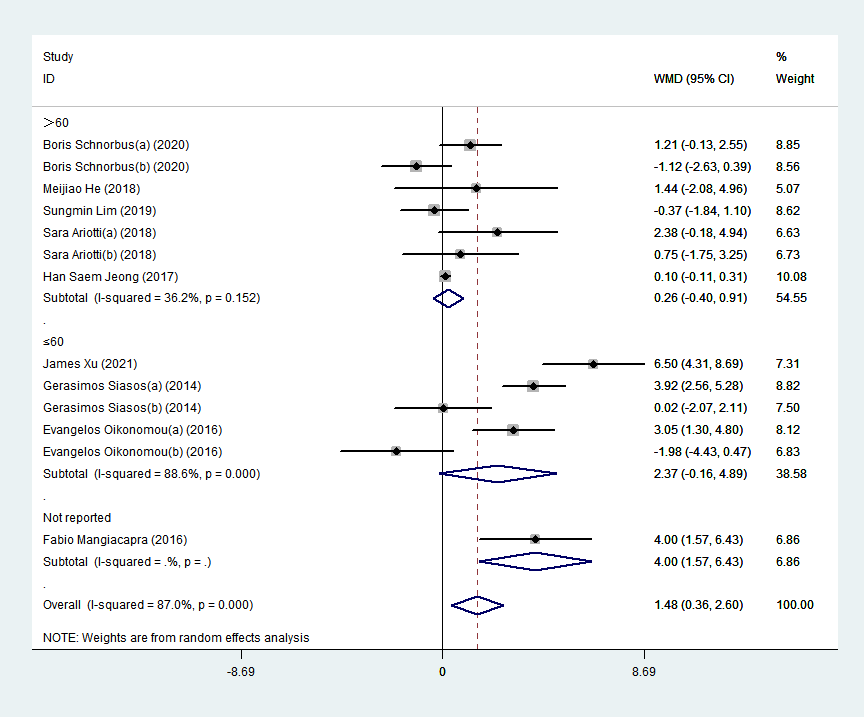


**Supplemental Figure 6: Pooled estimate of ticagrelor effect on reactive hyperemia index by subgroup according to controlled administration**


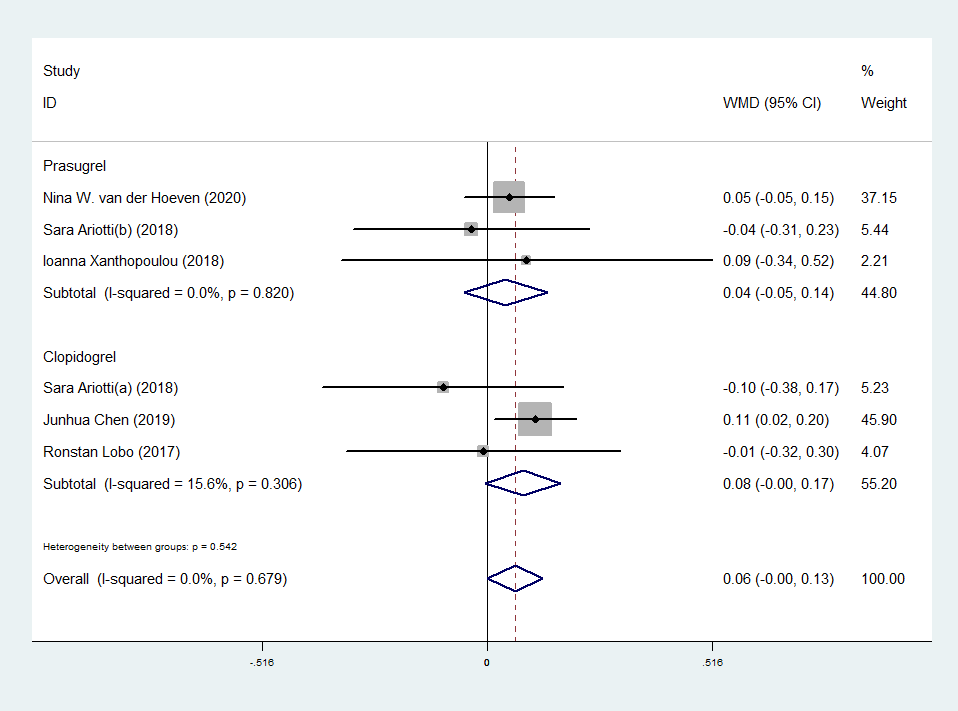


**Supplemental Figure 7: Pooled estimate of ticagrelor effect on reactive hyperemia index by subgroup according to study design**


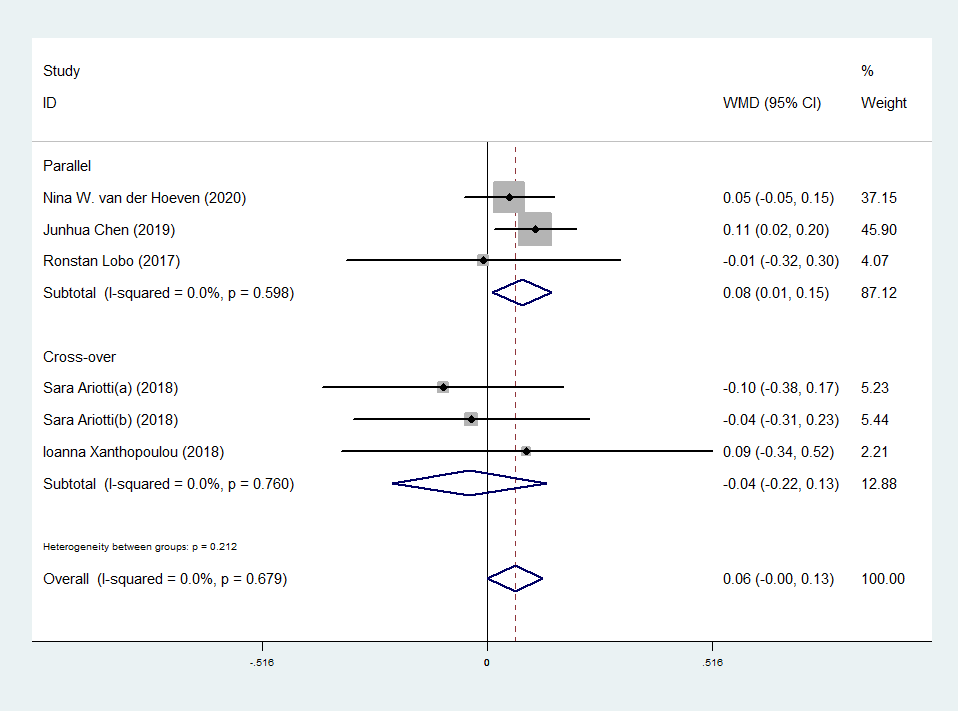


**Supplemental Figure 8: Pooled estimate of ticagrelor effect on reactive hyperemia index by subgroup according to study duration**


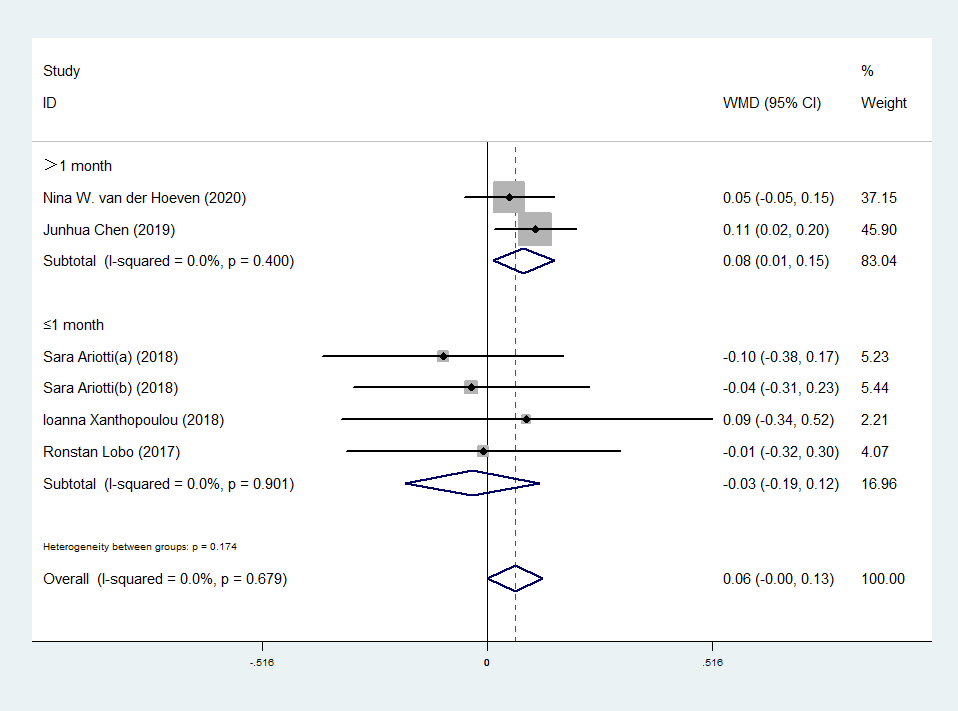


**Supplemental Figure 9: Publication bias assessment by used Egger's test for flow-mediated dilation**

**Supplemental Figure 10: Publication bias assessment by used Egger's test for reactive hyperemia index**

**Supplemental Figure 11: Publication bias assessment by used Egger's test for index of microvascular resistance**
